# Literature meta-analysis of zosteriform cutaneous metastases from melanoma and a clinico-histopathological report from India

**DOI:** 10.3332/ecancer.2013.324

**Published:** 2013-06-11

**Authors:** Savita Chaudhary, Cherry Bansal, Ayanat Husain

**Affiliations:** 1 Department of Dermatology, Era’s Lucknow Medical College and Hospital, Lucknow, Uttar Pradesh, India; 2 Department of Pathology, Era’s Lucknow Medical College and Hospital, Lucknow, Uttar Pradesh, India; 3 Department of Surgery, Era’s Lucknow Medical College and Hospital, Lucknow, Uttar Pradesh, India

**Keywords:** carcinoma, skin neoplasm, secondary, melanoma, herpes zoster, meta-analysis

## Abstract

Cutaneous metastases in patients with malignant tumours are an important clue to tumour progression or even the first manifestation of malignancy. Among the various topographic patterns of cutaneous metastasis, the zosteriform pattern of metastasis is rare, and very few cases have been published. Various theories have been proposed for this zosteriform pattern of metastases, but none have been proved. We collected the available PubMed articles on zosteriform skin metastasis from cutaneous melanoma published since 1970 and reviewed the cases, including our own case. Melanoma presents with cutaneous metastasis in up to 44% of patients. Clinicians treating oncology patients should consider zosteriform skin metastasis in the differential diagnosis of zosteriform lesions to avoid inadequate diagnosis and management.

## Introduction

Skin metastases are very common among patients with melanoma. Skin is involved as the site of metastasis in approximately 10% of metastases from all primary neoplasms, and up to 44% of malignant melanoma has cutaneous metastasis [1].

From a clinical point of view, the most common presentations of cutaneous metastatic disease are papules and nodules, solitary or widespread, sometimes ulcerated. However, a wide morphological spectrum of lesions has been described, including erythematous patches or plaques, inflammatory erysipela-like lesions, diffuse sclerodermiform lesions with induration of the skin (‘en cuirasse’ metastatic carcinoma), telangiectatic papulovesicles, purpuric plaques mimicking vasculitis, and alopecia areata-like scalp lesions [1–4]. Malignancies with zosteriform cutaneous metastases originate in skin, blood vessels, blood, and viscera [5].

The zosteriform pattern has been described in few cases; a recent meta-analysis reviewed 56 cases published in the English literature since 1970 [6]. Several theories have been proposed in the literature to explain the pathogenetic mechanism of zosteriform dissemination, but none have been adequately proved.

In this article, we report a case of cutaneous melanoma with zosteriform metastasis, and we meta-analyse the available literature about zosteriform metastasis secondary to melanoma. The aim is to detail the morphological pattern of zosteriform metastasis secondary to melanoma.

## Case Report

A 70-year-old male presented to the dermatology department of Era’s Lucknow Medical College, Lucknow, India, with a chief complaint of painful nodular lesions progressively increasing in size and number over the medial and anterior aspect of the thigh since one month before.

**Figure 1:**
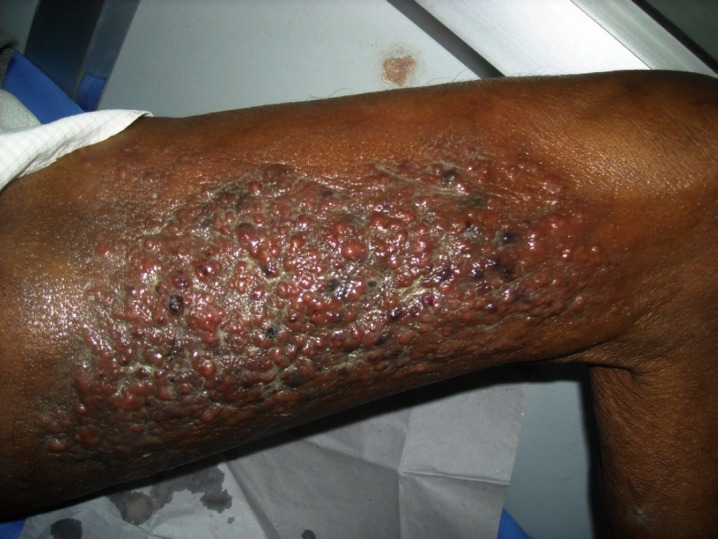
Left thigh medial aspect showed multiple papulonodular lesions that coalesced to form a plaque in dermatomal segment L2–L3.

On examination of the medial aspect of the left thigh, multiple papulonodular lesions of sizes varying from 0.5 cm × 0.5 cm to 1 cm × 2 cm were seen in dermatomal segment L2–L3, which coalesced to form an indurated plaque of size 10 cm × 15 cm with an irregular, erythematous surface and an ill-defined border (Figure 1).

An examination of the inguinal region revealed multiple discrete, firm, non-tender, mobile lymph nodes on the left side varying from 1 to 4 cm in size. Fine needle aspiration cytology smears from the lymph nodes showed melanoma cells with occasional pigmentation.

**Figure 2:**
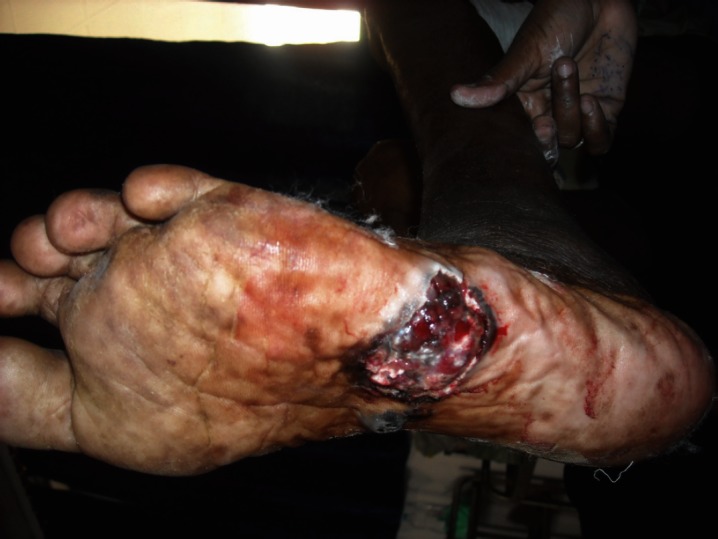
The sole of the left foot showed a larger ulcerated, ill-defined, pigmented lesion of size 4 cm × 5 cm, present laterally, and a smaller pigmented firm plaque of size 2 cm × 3 cm with well-defined margins and a raised surface, present medially.

On complete examination of the patient, two non-tender lesions were found on the sole of the left foot (Figure 2). One was an ulcerated, ill-defined, pigmented lesion of 4 × 5 cm in size, present laterally on the middle part, and the other was a pigmented firm plaque of 2 cm × 3 cm in size with well-defined margins and a raised surface. These lesions had been present for one year and were progressively increasing in size, and the larger lesion had ulcerated one month before the examination.

A provisional diagnosis of herpes zoster left thigh L2–L3 was considered, but the history did not corroborate with the disease. The lesion on the left thigh was not vesiculobullous, the plaque was firm on palpation, and the corresponding lymph nodes were also enlarged, firm, and multiple. Diagnostic punch biopsies from the left thigh plaque and the sole lesion were done. The tissue bled profusely and was very friable.

**Figure 3:**
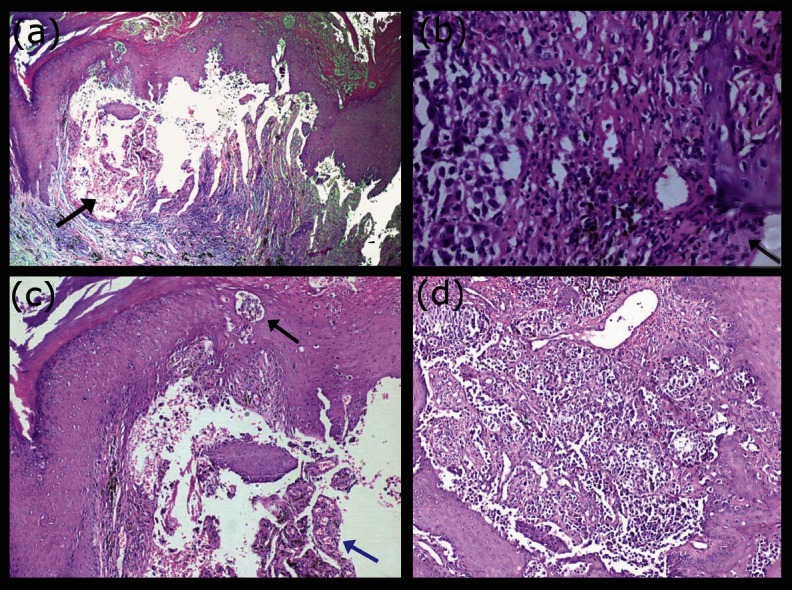
a. Photomicrograph from the primary origin ulcerated site simulated acantholytic bullous lesion in the scanner view (H & E; 100×). b. Photomicrograph exhibiting neutrophilic and fibrinous exudate toward the ulcerated surface (arrow). Tumour cell nests are present deep in the reticular dermis (H & E; 200×). c. Proliferation of single atypical pigmented melanocytes and nests (black arrow) seen in the epidermal layers. Increase in dermal blood vessels with proliferating new vessels present in the dermis (blue arrow) (H & E; 100×). d. Photomicrograph showing dense infiltration of melanoma cells with pigment at places. Atypical cells have descended deep into the reticular dermis (H & E; 100×).

A histopathological examination from the primary site with clinically evident ulcerated surface showed a lesion simulating acantholytic bulla in the scanner view (Figure 3a). The keratin layer thickness of the plantar surface was normal and exhibited evidence of ulceration with neutrophilic and fibrinous exudation toward the surface (Figure 3b). Proliferation of single atypical pigmented melanocytes was seen in the epidermal layers. The single melanocytes and the cell nests were distributed as runs of the cells in the papillary and deep reticular dermis (Clark level IV lesion). Asymmetrical inflammatory infiltrate of lymphocytes was present around tumour nests and showed crushing artefact. An increase in dermal blood vessels with proliferating new vessels was present in the dermis (Figure 3c). The maturation sequence for melanocytes as they descend into the dermis was not very evident. Melanoma cells were seen as large cells with abundant eosinophilic cytoplasm, vesicular nuclei, and very large, eosinophilic nucleoli.

The haematoxylin and eosin-stained section from the non-ulcerated area of the primary site showed architectural changes within the epidermis, with poor circumscription of melanocytes, which were present as intra-epidermal nests and single cells beyond the basal level, often exhibiting cytoplasmic melanin pigment (Figure 3d). The melanocytes had haphazard and aberrant distribution in the dermis, with a perivascular arrangement at some places. The tumour cells had a large, irregular nucleus with vesicular chromatin, prominent eosinophilic nucleoli and visible intracellular melanin pigment. Brisk mitotic activity was present.

**Figure 4:**
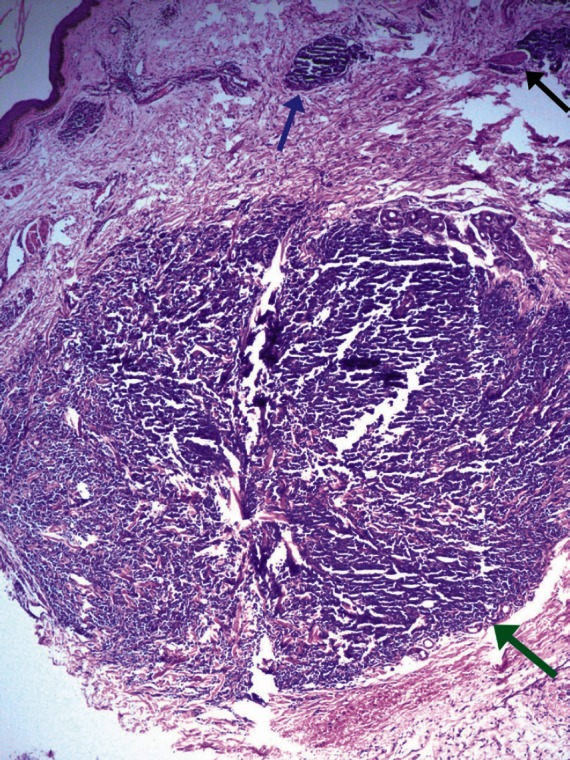
Photomicrograph from the metastatic site showing a circumscribed tumour nodule of hyperchromatic tumour cells in dermis with no junctional activity (green arrow). A small tumour nest (blue arrow) and perineural tumour focus (black arrow) are seen in upper dermis (H & E; 100×).

The histological appearance of melanoma metastasis in the thigh skin differed from that of the primary melanoma site by the absence of junctional activity, non-involvement of the epidermis, absence of inflammatory infiltrate, and different tumour cell morphology. There was little tendency for maturation of melanocytes with progressive descent through the dermis. Nests of atypical cells were seen in the reticular and deep dermis. Dermal invasion was characterised by proliferation of hyperchromatic melanocytes in fairly symmetrical small and large cellular nodules. The pattern to aggregate around eccrine structures and small blood vessels was clearly appreciable. A focus of perineural aggregate was also seen in the section. However, the nodules did not involve the subcutis included in the section examined. Cytologically, unlike the primary site, the cells were uniformly atypical with hyperchromatic, irregular nuclei with dense nuclear chromatin and inconspicuous nucleoli. No melanin pigment was visible in the melanocytes at the metastatic site. Mitotic activity was low and very occasional mitotic figure was seen. The overlying epidermis was largely unremarkable (Figure 4). Immunohistochemistry for HMB-45 was positive in tumour cells.

On the basis of clinical presentation and histopathological examination, the case was diagnosed as melanoma of the left sole with zosteriform cutaneous metastasis.

A literature meta-analysis of melanoma cases with zosteriform metastases was performed.

We reviewed the literature from Pubmed published since 1970 and found very few reported cases of melanoma with zosteriform cutaneous metastases. To the best of our knowledge, no case report has been published from India. To date, 12 cases have been reported, including our case, as shown in Table 1. Out of the 12 patients, nine were males and three were females, that is, 75.0% males and 25.0% females. Most of the patients were in the sixth and seventh decade of life. The site of melanoma varied widely: pre-auricular (one case), shoulder (one case), interscapular (one case), chest (one case), back (three cases), lumbar region (two cases), right hip (one case), unknown primary (two cases), and sole in the present case, and this is the first case of melanoma of the sole ever reported having zosteriform cutaneous metastasis. The time from the first diagnosis to zosteriform metastasis varied from simultaneous (as in our case) to five years. The sites of metastatic skin lesions as described in previous studies were the scalp (one case), chest (three cases), forearm (one case), trunk (six cases) and back (one case), and thigh in our case. Vesiculobullous lesions were present in six cases (50%), but in our case, multiple nodular lesions were seen, coalescing to form a plaque. Metastatic involvement with regional lymph nodes was seen in 8 out of 12 cases including the present case (66.7%). The overall patient outcome was poor, the mean survival from skin involvement ranged from 1 to 36 months. Cutaneous melanomas account for 19.05% (12/63) of zosteriform metastases reported in the literature. Zosteriform localisations usually arise in the same topographic locations as the primary melanoma.

## Discussion

Skin metastases appear in 10% of cancer patients [1]. Melanoma presents with skin metastasis (up to 44% cases) [1], and up to 1% of patients presenting with metastasising melanoma do not have a detectable primary tumour [7, 14]. Many different malignant tumours can metastasise to the skin, but the most common primary sources are the breast, stomach, lung, and uterus [13]. These lesions usually present as firm papules or nodules, both of which may ulcerate; occasionally, they are inflammatory, sclerotic, bullous or vesicular.

Topographically cutaneous lesions may present at any site near or far from the area of primary malignancy, but zosteriform cutaneous metastasis is a rare occurrence. Zosteriform metastasis may be painful, tender, or pruritic and consists of vesicles on a background of erythema, imitating the appearance of shingles. The vesicles are commonly confined to single unilateral dermatome, adding to the potential of misdiagnosis. Paola Savoia *et al *[6] analysed 56 cases of zosteriform metastases secondary to various tumours in 2009. We reviewed PubMed articles related to zosteriform cutaneous metastases and found six more cases of zosteriform metastases reported by various authors to date. To the best of our knowledge, 63 cases (including our case) of zosteriform cutaneous metastasis have been reported, out of which 12 (19.05%) were secondary to melanoma [15–20]. Several theories have been proposed to explain the mechanism of the zosteriform distribution of metastases. Some of the patients described in the literature had a history of a zoster infection in the same dermatome in which metastatic lesions were subsequently observed [8, 21–26]. In these cases, the zosteriform pattern could be a consequence of a Koebner or Koebner-like phenomenon in a site of diminished resistance of the skin [8, 23, 24]. More recently, it has been suggested that neural alteration caused by the herpes virus resulted from an impairment of the immunological function of the overlying skin that, consequently, could be more receptive to metastatic cell homing [27]. Several authors showed evidence of viral DNA from the skin of patients with zosteriform metastases [8, 21–26], and they did not refer to previous varicella zoster virus infections. Other possibilities to explain the spread of tumour cells with a zosteriform pattern include a direct invasion from underlying structures (in the case of internal cancer) [28], surgical implantation of neoplastic cells into the skin [29], and invasion of the perineural lymphatic vessels or the dorsal root ganglion fenestrated vasculature. The clinical evidence, reported by several authors, of tissue swelling and the histopathologic demonstration of enlarged lymphatic vessels with focal neoplastic embolism support the hypothesis of metastatic spread through the lymphatic system [15, 30, 31]; widespread lymphatic obstruction by tumour cells can result in retrograde flow that spreads malignant cells into the skin [11]. In our opinion, this last hypothesis seems more apt to describe the pathogenesis of zosteriform metastases in our patient. In fact, all these patients demonstrated involvement of regional lymph nodes, with concomitant development of cutaneous metastases. In summary, cutaneous metastases with zosteriform pattern are rare entities, and among these, ones due to melanoma are the rarest. Hence, the published data about zosteriform melanoma metastases are too scanty and incomplete to minutely define this entity [6]. In our case, histopathological examination played an important role in making the diagnosis and ruling out herpes zoster.

## Conclusion

In patients presenting with melanoma and lesions of zosteriform pattern, an accurate medical history could be of primary importance for the right classification of the disease and for treatment choice. Moreover, clinicians treating oncology patients should consider this rare form of cutaneous involvement in the differential diagnosis of zosteriform lesions to avoid inadequate diagnosis and management.

## Figures and Tables

**Table 1:** Review of melanoma cases presenting with zosteriform metastasis.

Author	Sex/age	Site of primary (melanoma)	Time from first diagnosis to zosteriform metastasis	Site of zosteriform metastasis	Vesiculobullous lesions	Metastatic involvement	Survival (from skin involvement)
Martinez [7]	M/85	Melanoma (primary unknown)	simultaneous	Buttocks hip, leg (L1–L2)	No	NA	1 month
Zalaudek [8]	F/59	Melanoma (4 mm back)	2 years	Trunk, back (D7–D9)	No	Regional nodes	NA
Stern [9]	F/53	Melanoma	NA	Forearm	NA	NA	1 year
Itin [5]	F/29	Melanoma (2.3 mm back)	<5 months	Trunk (T5)	No	Regional nodes	17 months
North [10]	M/63	Melanoma (5 mm preauricular)	5 years	Trunk (T12)	Yes	Regional nodes	3 months
Kondras [11]	M/65	Melanoma (11 mm back)	2 months	Back	Yes	Regional nodes	NA
Galindo [12]	M/79	Melanoma (4 mm chest)	11 months	Chest (T6)	No	No	NA
Evans [13]	M/73	Melanoma (1.25 mm shoulder)	5 years	Scalp	Yes	NA	NA
Paola Savoia [6]	M/72	Melanoma (16 mm lumbar region)	3 months	Chest (T2–T8)	Yes	Regional nodes	6 months
Paola Savoia [6]	M/81	Melanoma (6 mm right hip)	4 months	Trunk (L1–L4)	Yes	Regional nodes	5 months
Paola Savoia [6]	M/63	Melanoma (2 mm and 1.3 mm inter-scapular region)	1 month	Chest (T4–T8)	Yes	Regional nodes	Alive (43 years)
Present Author	M/72	Melanoma (4.5 mm and 4 mm sole)	simultaneous	Thigh (L2–L3)	No	Regional nodes	1 month

F-Female, M-Male, NA-Not available, D-Dorsal segment, T-Thoracic segment, L-Lumbar segment
